# Supplementary material for: Comprehensive Assessment of Subtyping Methods for Improved Surveillance of Foodborne Salmonella
Source: Microbiol Spectr. 2022 Oct 4;10(5):e02479-22. doi: 10.1128/spectrum.02479-22 (PMC9602514; doi:10.1128/spectrum.02479-22)
Supplement: Supplemental file 1 — Fig. S1 to S4. Download spectrum.02479-22-s0001.pdf, PDF file, 0.7 MB [file spectrum.02479-22-s0001.pdf]

Supplemental Material

Table S1 Typing results, isolation information of 61 isolates used in this study

Table S2 Two CRISPR loci's spacer sequence data and information

Table S3 CRISPR-MVLST sequence types (CSTs) of the studied *Salmonella* isolates (n=61)

Table S4 wgMLST profile matrix of the studied *Salmonella* isolates (n=61)

Table S5 Information of samples from different stages of the poultry production chain

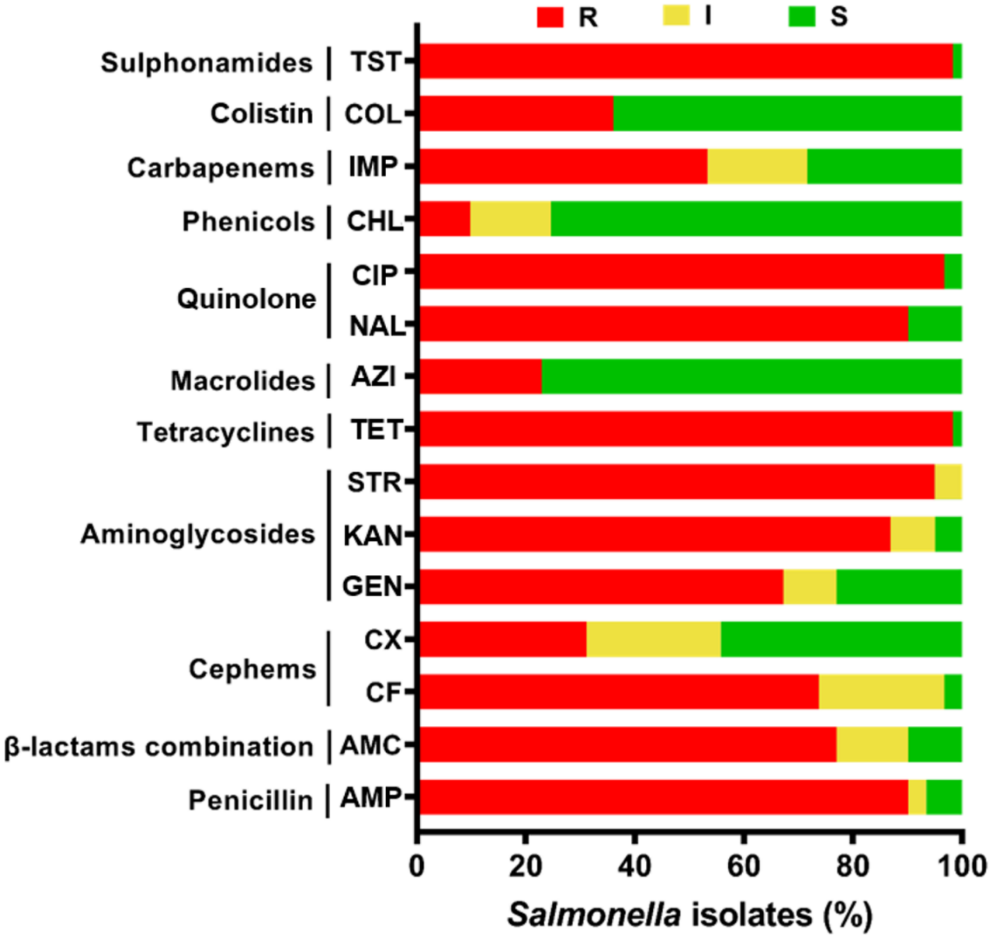

Figure S1 Antimicrobial susceptibility results of the studied *Salmonella* isolates (n=61). The X-axis shows the percentage of isolates showing antimicrobial resistance (AMR) phenotype. The Y-axis shows 15 antimicrobial molecules of 11 different classes. The red bar represents percentage of isolates showing AMR among the selected 61 isolates from the entire poultry production chain. The yellow bar indicates the intermediate resistance, and the green bar indicates the susceptibility of isolates.

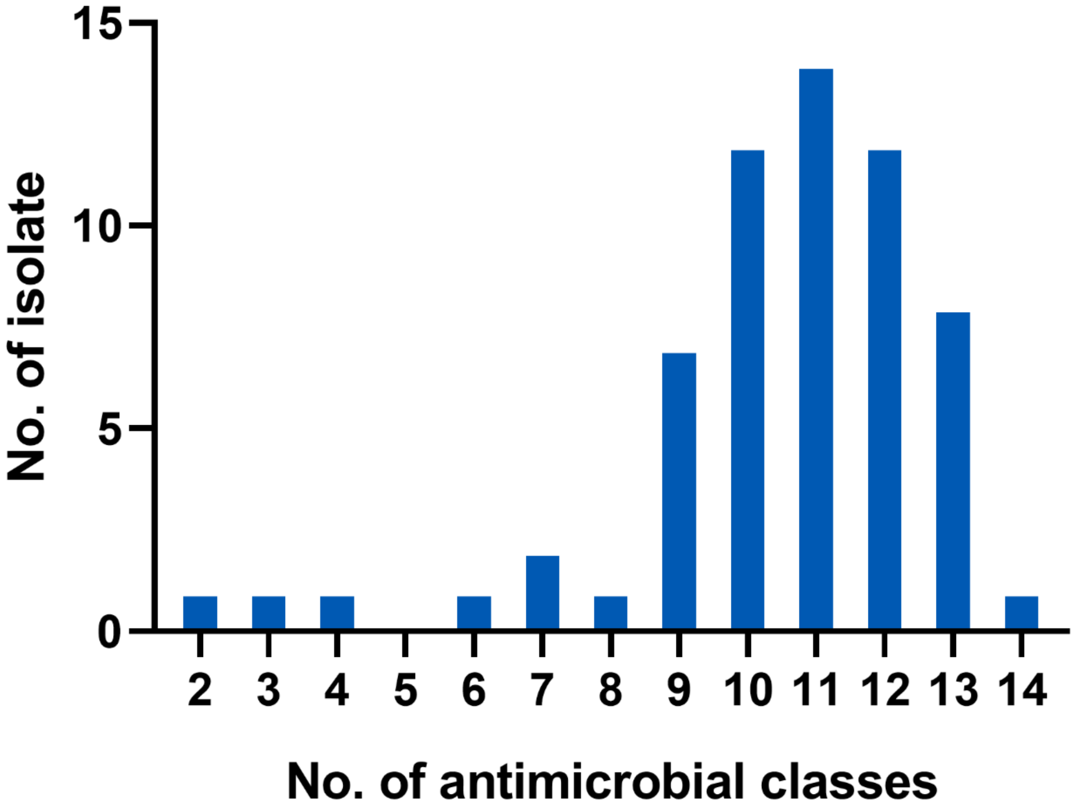

Figure S2 Number bacterial isolate with multiple antimicrobial resistance. The X-axis shows the number of antimicrobial resistance classes found in one isolate. The Y-axis shows the number of isolates, in which the corresponding multiple resistance was detected out of all 61 isolates from the poultry production chain.

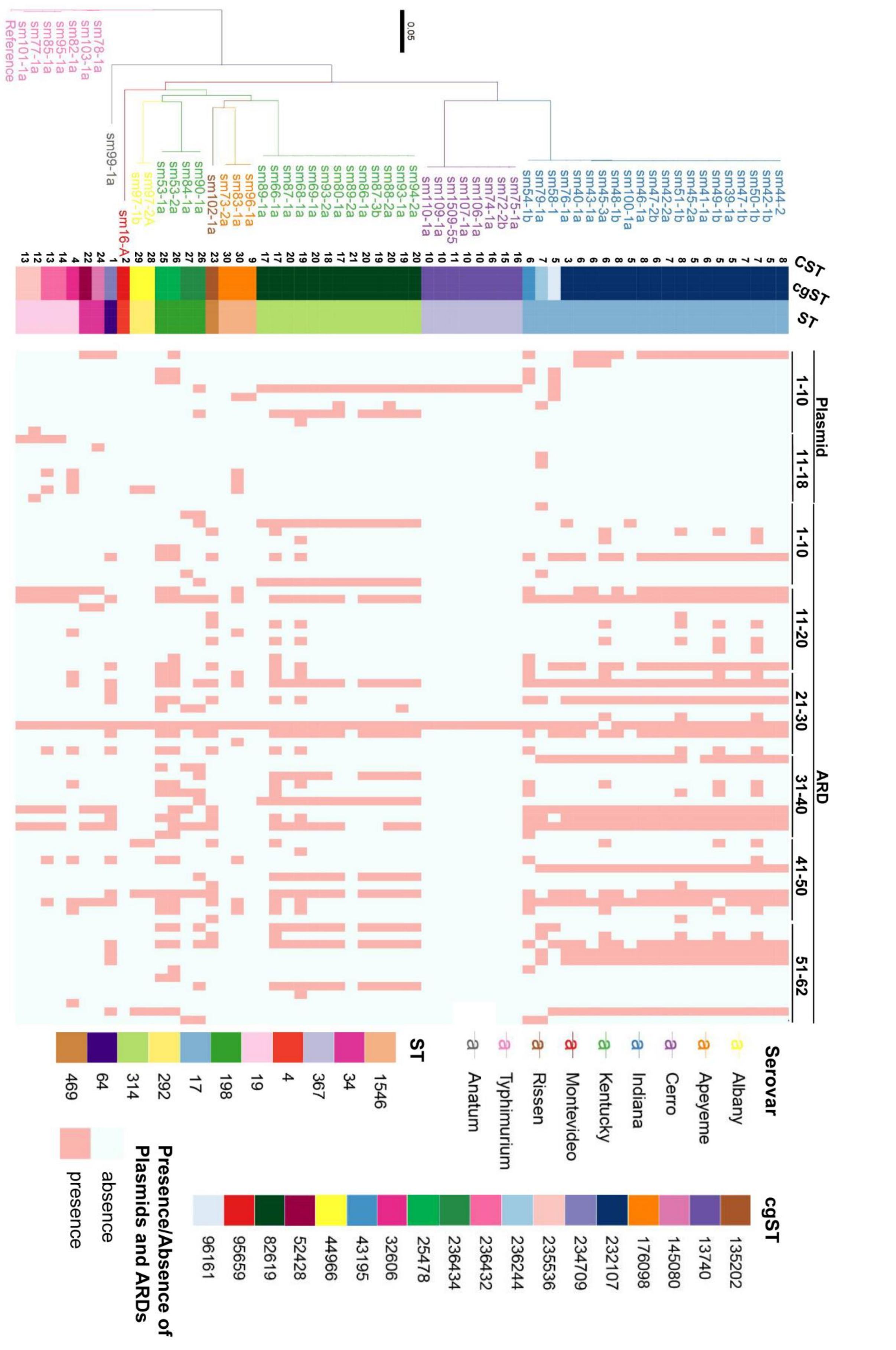
0.05
sm44-2
sm42-1b
sm50-1b
sm47-1b
sm49-1b
sm39-1a
sm41-1a
sm45-2a
sm51-1b
sm42-2a
sm47-2b
sm46-1a
sm100-1a
sm48-1b
sm45-3a
sm43-1a
sm40-1a
sm76-1a
sm58-1
sm79-1a
sm54-1b
sm75-1a
sm72-2b
sm74-1a
sm106-1a
sm107-1a
sm1509-55
sm109-1a
sm110-1a
sm94-2a
sm93-1a
sm88-2a
sm87-3b
sm86-1a
sm89-2a
sm80-1a
sm93-2a
sm69-1a
sm68-1a
sm87-1a
sm66-1a
sm89-1a
sm96-1a
sm83-1a
sm73-2a
sm102-1a
sm90-1a
sm84-1a
sm53-2a
sm53-1a
sm87-2A
sm97-1b
sm16-A
sm99-1a
sm78-1a
sm103-1a
sm82-1a
sm95-1a
sm85-1a
sm77-1a
sm101-1a
Reference
CST
cgST
ST
Plasmid
1-10
11-18
ARD
1-10
11-20
21-30
31-40
41-50
51-62
Serovar
Albany
Apeyeme
Cerro
Indiana
Kentucky
Montevideo
Rissen
Typhimurium
Anatum
ST
1546
34
367
4
19
198
17
292
314
64
469
cgST
135202
13740
145080
176098
232107
234709
235536
236244
236432
236434
25478
32606
43195
44966
52428
82619
95659
96161
Presence/Absence of Plasmids and ARDs
absence
presence

Figure S3 Results of *Salmonella* typing, plasmid and antimicrobial resistance determinant (ARD) detection among 61 isolates. A phylogenetic tree was projected for showing the genetic relationship of 61 isolates and the visualization of core genome SNP typing. Different serotypes were labeled with different colors as shown in the right part near the heatmap. A parallelly matched heatmap that was aligned to the phylogenetic tree was drawn. The left part of the heatmap shows the CST, cgST and ST. Different colors are used to distinguish different types as noted in the right part of the figure. The right part shows a detailed matrix of plasmids and ARDs (including AMR gene and AMR chromosome mutations). If a plasmid or an ARD is present in an isolate, the corresponding block is marked as pink. Otherwise, it is marked as light blue. The name of each plasmid or ARD is listed here:

Plasmid 1-10: "IncQ1","Col156","IncHI2","IncHI2A","IncX1", "IncFII(p96A)","IncN","IncR","Col3M","IncFIB(S)"

Plasmid 11-18: "IncFII(S)","p0111","IncA/C2","IncY","IncHI1A", "IncHI1B(R27)","IncI1","IncFIB(S)"

ARD 1-10: "*bla*$_{CTX-M-14}$","*bla*$_{CTX-M-14b}$","*bla*$_{CTX-M-55}$","*bla*$_{CTX-M-65}$","*bla*$_{CMY-2}$", "*bla*$_{DHA-1}$","*bla*$_{OXA-1}$","*bla*$_{TEM-105}$","*bla*$_{TEM-116}$","*bla*$_{TEM-141}$",

ARD 11-20: "*bla*$_{TEM-1B}$","*tet(A)*","*tet(B)*","*tet(C)*","*tet(J)*", "*tet(M)*","*cat*","*cat1*","*catA1*","*catB3*",

ARD 21-30:”*cmlA1*”,”*floR*”,”*aac(6')-aph(2'')*”,”*aac(3)-IVa*”,”*aac(3)-Id*”, “*aac(3)-IId*”,”*aac(6')-Iaa*”,”*aac(6')-Ib-cr*”,”*aadA1*”,”*aadA2*”

ARD 31-40:”*aadA5*”,”*aadA7*”,”*aadA16*”,”*ant(3'')-Ia*”,”*aph(3')-Ia*”, “*aph(3')-IIa*”,”*aph(3'')-Ib*”,”*aph(4)-Ia*”,”*aph(6)-Id*”,”*rmtB*”,

ARD 41-50:”*dfrA1*”,”*dfrA10*”,”*dfrA12*”,”*dfrA17*”,”*dfrA27*”, “*dfrA32*”,”*sul1*”,”*sul2*”,”*sul3*”,”*ere(A)*”,

ARD 51-62:”*mph(A)*”,”*fosA3*”,”*ARR-3*”,”*oqxA*”,”*oqxB*”, “*qepA1*”,”*qnrB4*”,”*qnrB6*”,”*qnrD1*”, “*qnrS1*”, “*gyrA*_S83F”,”*gyrA*_S83F+D87G”

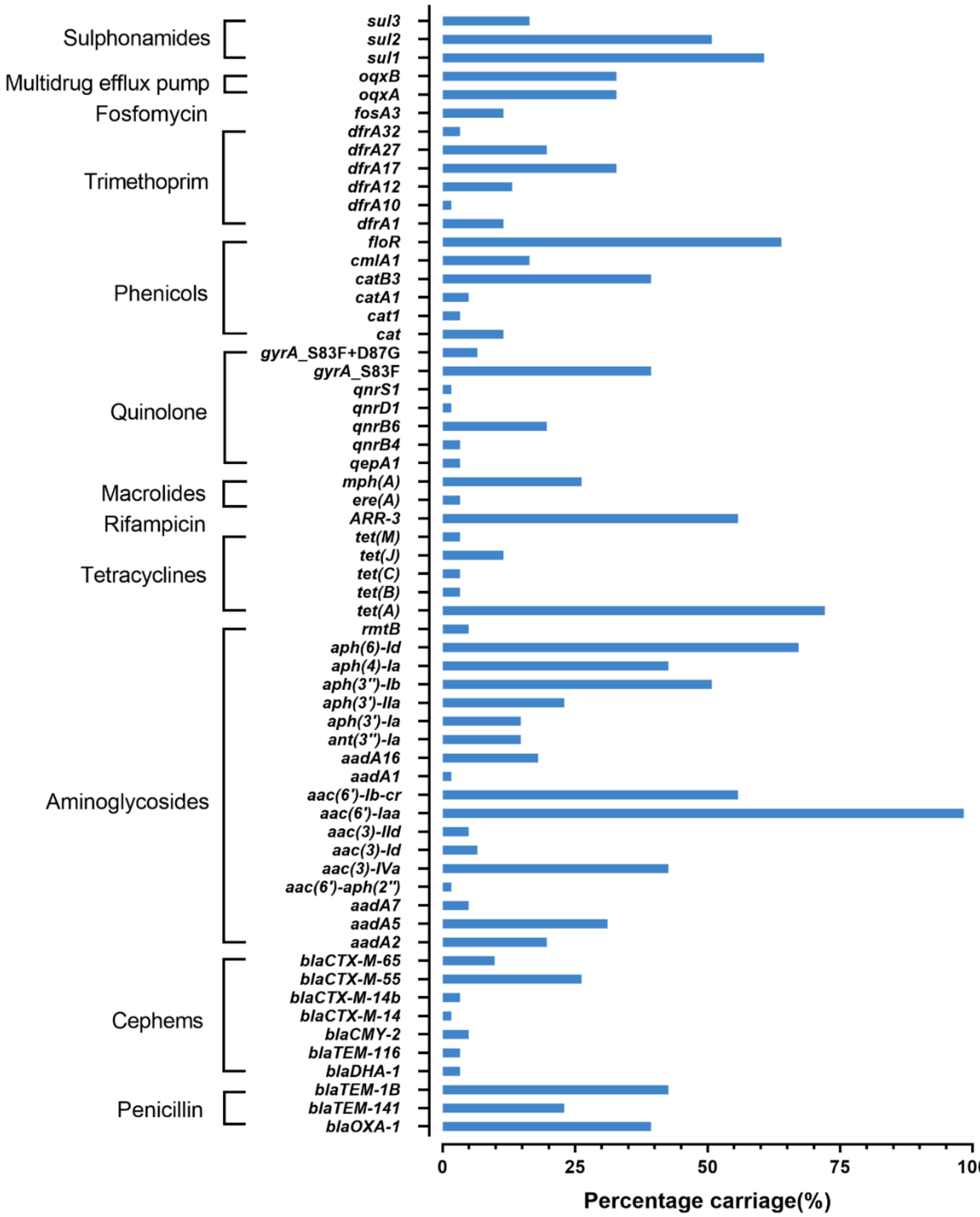

Figure S4 Detection of antimicrobial resistance determinants. The X-axis shows the percentage of isolates carrying specific antimicrobial resistance (AMR) genes. The Y-axis shows all detected AMR genes for nine classes of antimicrobials. The bar in blue represents AMR gene-carrying isolates from 61 isolates in this study.
